# Low complexity beamforming design in reconfigurable intelligent surface-assisted communication systems

**DOI:** 10.1038/s41598-024-63873-4

**Published:** 2024-06-11

**Authors:** Jun Jiang, Huan Huang, Junxin Zhang

**Affiliations:** 1 College of Information Science and Technology, Tibet University, Lhasa, 850000 China; 2 Information Technology National Experimental Teaching Demonstration Center of Tibet University, Lhasa, 850000 China; 3 Tibet University Tibetan Information Technology Engineering Research Center of the Ministry of Education, Lhasa, 850000 China

**Keywords:** Electrical and electronic engineering, Information technology

## Abstract

Due to the possibility of blockage during communication, the communication quality may be poor. Therefore, Reconfigurable Intelligent Surface (RIS) technology can effectively solve this problem. In order to increase the capacity of RIS-assisted wireless communication systems, joint optimization of beamforming design is crucial. However, the complexity of the optimization algorithm increases with the increase in the number of base station antennas and RIS elements deployed. Therefore, we propose a joint low-complexity optimization beamforming design based on fractional programming (FP) to address this issue. Specifically, we first use perfect channel state information to maximize the system's sum rate, as this problem is non-convex, we decompose the original problem into three sub-problems, and then introduce appropriate auxiliary variables. We derive optimal closed-form solutions for active and passive beamforming types, respectively. As the optimal solution obtained leads to higher computational complexity with an increase in the number of base station antennas and RIS elements, we reduce the computational complexity of obtaining the optimal solution based on the Woodbury transformation and scalar transformation. The proposed algorithm is also extended to the case where there is channel state information error. Finally, simulation results show that the proposed algorithm has certain advantages over other algorithms.

## Introduction

In fact, RIS consists of many elements that can manipulate incident signals through passive beamforming. Reconfigurable Intelligent Surface (RIS) is considered a promising technology for future 6G wireless communication systems^1–3^. RIS is a surface with numerous passive reflecting elements that can induce phase shifts in the transmitted signals^4–8^. By adjusting the phase shifts of these elements, the signal paths can be dynamically controlled, thus avoiding weak signal conditions in the presence of communication obstacles. Since RIS does not require signal processing or RF chains^9^, it does not introduce additional noise during communication, thereby enhancing system capacity and energy efficiency^10–12^. Therefore, due to these reasons, RIS has attracted increasing research interest in areas such as sensing integration^13,14^ physical layer security^15^, and terahertz communications^16,17^. Among them, achieving high system capacity and rate to improve user service quality is crucial for RIS-assisted wireless communication systems^18–20^.

For RIS-assisted wireless communication systems, the design and low-cost deployment present new challenges. Maximizing the communication system gain by jointly optimizing the active beamforming matrix of the base station (BS) and the reflection coefficients of RIS is an important problem^21,22^. Furthermore, traditional joint beamforming optimization algorithms^6,18^ become computationally complex and time-consuming when the number of BS antennas and RIS elements increases. Therefore, to address these issues, this paper proposes a low-complexity design for active beamforming at the transmitter and passive beamforming at RIS in RIS-assisted wireless communication systems. Additionally, the more practical scenario of channel estimation errors is considered since perfect channel state information is difficult to obtain in practical communication scenarios. Thus, the low-complexity joint beamforming algorithm is extended to incorporate realistic channel state information.

Recently, a significant amount of work has been done on joint beamforming optimization in RIS-assisted communication systems. For instance, in Ref.^23^, the optimization problem is characterized by limiting the transmit power of a single base station and RIS, followed by the adoption of a semi-definite relaxation algorithm to optimize the reflection coefficients with high complexity. In Ref.^24^, in a multi-RIS-assisted downlink communication system, the authors formulate an optimization problem for minimizing mean square error (MSE) and employ a maximum-minimum alternating optimization algorithm. By considering the communication interference caused by different bandwidths in multiple sub-intervals and the frequency-selective characteristics of RIS, the joint optimization problem is transformed into several sub-problems using fractional programming (FP)^25^ and block coordinate descent, respectively solved by maximum-minimum (MM) algorithm and alternating direction method of multipliers (ADMM)^26^. In the scenario of RIS-assisted communication with multiple base stations and users, the problem of determining which base station and user the RIS should serve is addressed using a deep reinforcement learning (DRL) algorithm^27^, which not only solves this problem effectively but also jointly optimizes beamforming.Considering that channel state information is generally not accurate in practical communication scenarios,^28^ investigates the optimization of RIS-assisted communication with imperfect channel state information (CSI) by maximizing the sum rate. In Ref.^29^, the authors consider the presence of noise in the reflection coefficients of RIS and transform the problem into minimizing the mean square error of data streams, which is solved using the MM algorithm. Furthermore, based on the constraint of constant modulus of RIS reflection coefficients, a modified Riemannian gradient ascent (RGA) algorithm is proposed. In Ref.^19^, a RIS-assisted non-orthogonal multiple access system is studied, aiming to maximize the system sum rate. By decomposing the optimization problem into three sub-problems, an alternating flow optimization algorithm and a continuous convex approximation algorithm are employed to jointly optimize the BS beamforming and RIS phase shifting. Based on the RIS-assisted massive MIMO downlink system without cellular coverage in Ref.^30^, the authors propose an FP-based approach to decompose the joint beamforming problem into two sub-problems and optimize them using the primal–dual subgradient (PDS) algorithm. In Ref.^31^, a RIS-assisted non-cellular IoT network is proposed, considering the large number of devices in the IoT architecture. To address the energy consumption issue associated with the traditional fully connected user-base station approach, a user-centric non-cellular network is designed, and a low-complexity RIS passive beamforming optimization algorithm is developed using the FP method. However, the active beamforming design still incurs high computational complexity. Therefore, in Ref.^32^, two time-scale channel state information is introduced for RIS passive beamforming optimization using statistical CSI and transmitter active beamforming optimization using instantaneous CSI, aiming to reduce overhead and improve communication efficiency in RIS-assisted non-cellular massive MIMO systems. In Ref.^33^, the authors exploited the duality between Gaussian MIMO broadcast channels and multiple access channels. They utilized alternating optimization algorithms to jointly optimize the user's covariance matrix and RIS phase shifts, deriving closed-form solutions for optimal performance and maximum system rates. In Refs.^34,35^, the authors investigated the non-linear coupling between elements of RIS in practical physical channel models. Subsequently, Ref.^36^ also confirmed that the assumed linear RIS cascaded channel model is inadequate for describing physical reality, thereby verifying the nonlinearity of RIS cascaded channels. Therefore, in this article, we assume that the cascaded channel is linear, which may lead to performance degradation in practical communication systems. Future work could explore more realistic RIS cascaded channel modeling and algorithm design to optimize system performance and rates.In this paper, we propose a low-complexity joint beamforming optimization algorithm for RIS-assisted communication systems. The main contributions are as follows:

- We propose a low-complexity joint beamforming optimization algorithm for RIS-assisted communication systems. Specifically, we formulate an optimization problem for maximizing the system sum rate based on perfect CSI in a multi-user RIS-assisted communication system. Since this problem is non-convex, we decompose the objective function into several sub-problems using the FP method and derive closed-form solutions for optimization.
- To address the high computational complexity issue of obtaining closed-form solutions as the number of base station antennas and RIS elements increases, we design a low-complexity joint optimization algorithm based on the "Woodbury transformation"^37^ and "scalarization transformation"^31^. The proposed low-complexity algorithm demonstrates significantly lower computational complexity compared to traditional optimization methods, even though multiple iterations are still required for convergence.
- In practical communication scenarios, perfect CSI is difficult to obtain. Therefore, we also consider the variation of maximizing the system sum rate under non-perfect CSI conditions. We extend the proposed low-complexity algorithm to the joint beamforming design under imperfect CSI. Simulation results show that the proposed low-complexity algorithm exhibits superior performance in large-scale MIMO systems and RIS with multiple elements compared to existing optimization algorithms.

The effectiveness of the proposed algorithm for joint beamforming design in RIS-assisted communication systems was tested through simulation results. In this article, the following notations were employed: italic denotes scalars, bold italic represents matrices or vectors, if $\cdot$ denotes a matrix, then $\text{diag}(\cdot )$ denotes a vector; if $\cdot$ denotes a vector, then $\text{diag}(\cdot )$ denotes a diagonal matrix. ${\left(\cdot \right)}^{T}$ denotes the transpose operation, ${\left(\cdot \right)}^{H}$ represents the conjugate transpose operation, ${\Vert \cdot \Vert }_{2}$ denotes the 2-norm, $\left|\cdot \right|$ denotes the magnitude, and ${\left(\cdot \right)}^{*}$ denotes the optimal solution.

## System model and problem formulation

In this section, we first describe the channel model, followed by the characterization of the signal model of the system. Finally, based on the given signal model, we design the formulation of the optimization problem.

**Figure 1:**
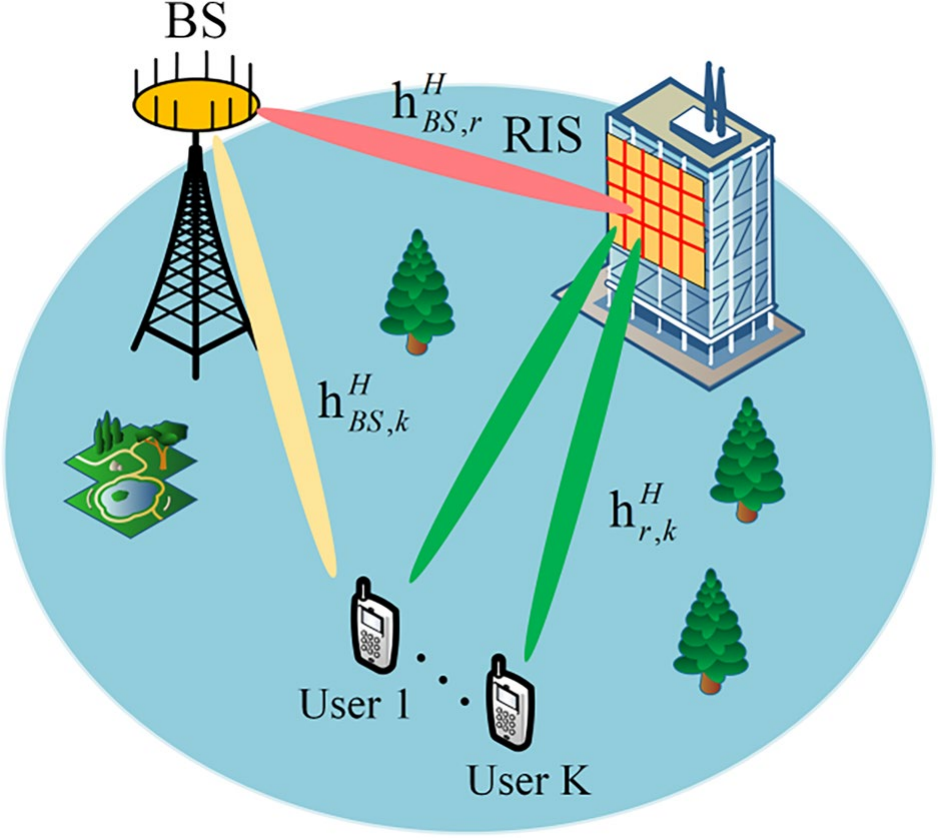
RIS-assisted multi-user communication system.

As shown in Fig. 1, we consider a RIS-assisted multi-user downlink communication system, where the base station is equipped with* M* antennas, there are *K* $\left(K<M\right)$ single-antenna users, and the RIS has* N* reflecting elements. The channels from user *K* to the base station, from the RIS to the base station, and from user *K* to the RIS are denoted as ${{\varvec{h}}}_{BS,k}\in {\mathbb{C}}^{M\times 1}$, ${{\varvec{h}}}_{BS,r}\in {\mathbb{C}}^{M\times N}\text{ and }{{\varvec{h}}}_{r,k}\in {\mathbb{C}}^{N\times 1}$, where $k=\left[1,\cdots ,K\right]$*.* The phase shift matrix of the RIS is denoted as $\widetilde{{\varvec{\theta}}}=diag\left({\widetilde{\theta }}_{1},\cdots ,{\widetilde{\theta }}_{n},\cdots {\widetilde{\theta }}_{N}\right)$, where ${\widetilde{\theta }}_{n}={\rho }_{n}{e}^{j{\phi }_{n}}$ denotes the reflection coefficient of the nth element on the RIS, while ${\phi }_{n}\in [\text{0,2}\pi )$ and ${\rho }_{n}\in [\text{0,1}]$ represent the phase shift and the on/off state of the nth element on the RIS, respectively. In the following, we determine the values of ${\rho }_{n}=1$ in order to achieve maximum system gain.

We assume that the direct link channel from the base station to the user in this paper follows Rayleigh fading, and the segmented channels from the base station to the RIS and from the RIS to the user follow Rician fading. The spacing configuration between the RIS elements and the base station antennas is a half-wavelength uniform linear array (ULA). Therefore, the channels ${{\varvec{h}}}_{BS,r}^{H}$ and ${{\varvec{h}}}_{r,k}^{H}$ are modeled as:

1 $${{\varvec{h}}}_{BS,r}^{H}={{d}_{h}}\left(\sqrt{{\frac{{\varpi }_{h}}{{{\varpi }_{h}}+1}}}{\overline{{\varvec{h}}} }^{H}+\sqrt{{\frac{1}{{{\varpi }_{h}}+1}}}{\widetilde{{\varvec{h}}}}^{H}\right),$$

2 $${{\varvec{h}}}_{r,k}^{H}={d}_{r,k}\left(\sqrt{\frac{{\varpi }_{r,k}}{{\varpi }_{r,k}+1}}{\overline{{\varvec{h}}} }_{r,k}^{H}+\sqrt{\frac{1}{{\varpi }_{r,k}+1}}{\widetilde{{\varvec{h}}}}_{r,k}^{H}\right),$$

where ${d}_{h}$ and ${d}_{r,k}$ represent the path loss between the base station and the RIS, and between the RIS and user $k$, respectively. ${\varpi }_{h}$ and ${\varpi }_{r,k}$ represent the Rician factors. ${\overline{{\varvec{h}}} }^{\text{H}}$ and ${\overline{{\varvec{h}}} }_{r,k}^{H}$ represent the line-of-sight (LoS) components of the channel paths. The response ${\overline{\mathbf{h}} }^{H}$ can be expressed using the ULA response of the $N$ elements of the base station antennas as:

3 $${{\varvec{c}}}_{M}\left({\vartheta }_{BS}\right)=\left[1,{e}^{j2\pi \frac{d}{\lambda }sin\vartheta }\cdots ,{e}^{j2\pi \frac{d}{\lambda }\left(M-1\right)sin\vartheta }\right],$$

where ${\vartheta }_{BS}$ represents the azimuth angle of the base station transmitted signal. Then,${\overline{\mathbf{h}} }^{H}$ can be expressed as:

4 $${\overline{{\varvec{h}}} }^{H}={{\varvec{c}}}_{N}^{H}({\vartheta }_{r}){{\varvec{c}}}_{M}\left({\vartheta }_{BS}\right),$$

where ${\vartheta }_{r}$ represents the angle at which the signal reaches the RIS. Based on the above derivation, ${\overline{{\varvec{h}}} }_{r,k}^{H}$ can be expressed as:

5 $${\overline{{\varvec{h}}} }_{r,k}^{H}={{\varvec{c}}}_{M}\left({\vartheta }_{k}\right),$$

where ${\vartheta }_{k}$ represents the angle at which the signal reaches user $k$. ${\widetilde{{\varvec{h}}}}^{H}\text{ and}{\widetilde{ {\varvec{h}}}}_{r,k}^{H}$ represent the NLoS components of the channel, where the elements follow complex Gaussian distribution with zero mean and unit variance $(\mathcal{C}\mathcal{N}\sim \left(\text{0,1}\right)).$ When modeling the signal, let ${s}_{k}$ represent the data symbol transmitted to the *K*-th user, where ${\mathbb{E}}[{s}_{k}{s}_{k}^{H}]=1$. Therefore, for all users, the signal ${\varvec{f}}$ transmitted from the base station can be expressed as:

6 $${\varvec{f}}=\sum_{k=1}^{K}{{\varvec{w}}}_{k}{s}_{k},$$

where ${{\varvec{w}}}_{k}\in {\mathbb{C}}^{M\times 1}$ represents the beamforming vector transmitted from the base station to the *K*-th user. Let ${{\varvec{H}}}_{k}={{\varvec{h}}}_{BS,k}^{H}+{{\varvec{h}}}_{r,k}^{H}\widetilde{{\varvec{\theta}}}{{\varvec{h}}}_{BS,r}^{H}$ represent all the channels that the signal received by user $k$ passes through. Therefore, the signal received by user $k$ can be expressed as:

7 $${y}_{k}={{\varvec{H}}}_{k}{\varvec{f}}+{n}_{k}={{\varvec{H}}}_{k}\sum_{k=1}^{K}{{{\varvec{w}}}_{\text{k}}}{{\text{s}}_{\text{k}}}+{n}_{k},$$

where ${n}_{k}\sim \mathcal{C}\mathcal{N}\left(0,{\upsigma }_{0}^{2}\right)$ represents the Gaussian white noise received by the $k$-th user. For the subsequent algorithm processing, let $\overline{{\varvec{\theta}} }=\left[{\widetilde{\theta }}_{1},\cdots ,{\widetilde{\theta }}_{n},\cdots {\widetilde{\theta }}_{N}\right]$. Then, we have:

8 $${{\varvec{h}}}_{r,k}^{H}{\overline{{\varvec{\theta}}} }^{H}{{\varvec{h}}}_{BS,r}^{H}={\overline{{\varvec{\theta}}} }^{H}diag\left({{\varvec{h}}}_{r,k}^{H}\right){{\varvec{h}}}_{BS,r}^{H}.$$

To express the equation more concisely, let ${{\varvec{h}}}_{a}=diag\left({{\varvec{h}}}_{r,k}^{H}\right){{\varvec{h}}}_{BS,r}^{H}\in {\mathbb{C}}^{N\times M}$. Then, Eq. (7) can be rewritten as:

9 $${y}_{k}=\left({{\varvec{h}}}_{BS,k}^{H}+{\overline{{\varvec{\theta}}} }^{H}{{\varvec{h}}}_{a}\right)\sum_{k=1}^{K}{{\varvec{w}}}_{k}{s}_{k}+{n}_{k}.$$

Therefore, The Signal-to-Interference-plus-Noise Ratio (SINR) of the user can be expressed as:

10 $$\begin{aligned}   {\gamma }_{k}&=\frac{{\left|\left({{\varvec{h}}}_{BS,k}^{H}+{\overline{{\varvec{\theta}}} }^{H}{{\varvec{h}}}_{a}\right){{\varvec{w}}}_{\text{k}}\right|}^{2}}{\sum_{j=1,j\ne k}^{K}{\left|\left({{\varvec{h}}}_{BS,k}^{H}+{\overline{{\varvec{\theta}}} }^{H}{{\varvec{h}}}_{a}\right){{\varvec{w}}}_{j}\right|}^{2}+{\sigma }_{0}^{2}}\\    &=\frac{{\left|{{\varvec{H}}}_{k}^{H}{{\varvec{w}}}_{\text{k}}\right|}^{2}}{\sum_{j=1,j\ne k}^{K}{\left|{{\varvec{H}}}_{k}^{H}{{\varvec{w}}}_{j}\right|}^{2}+{\sigma }_{0}^{2}}\end{aligned},$$

where ${{\varvec{H}}}_{k}^{H}=\left({{\varvec{h}}}_{BS,k}^{H}+{\overline{{\varvec{\theta}}} }^{H}{{\varvec{h}}}_{a}\right)$. Therefore, the sum rate of the system can be expressed as:

11 $${R}_{sum}=\sum_{k=1}^{K}{R}_{k}=\sum_{k=1}^{K}\text{log}\left(1+{\gamma }_{k}\right).$$

The purpose of this paper is to design optimization algorithms to maximize the sum rate of the system. The next step is to formulate the optimization problem. Based on the previous discussion, the objective function of the optimization problem has been determined. Now, let's discuss the constraints. Among them, the beamforming vector ${{\varvec{w}}}_{k}$ is related to the transmit power of the base station. Therefore, we have:

12 $$\sum_{k=1}^{K}{\Vert {{\varvec{w}}}_{k}\Vert }^{2}\le P,$$

where $P$ represents the total power transmitted by the base station. In addition, in order to achieve the maximum system gain, the constraints of the RIS elements may be expressed as:

13 $$\left|{\overline{\theta }}_{n}\right|=1, \forall n=1,\cdots ,N.$$

Let ${\varvec{W}}=\left[{{\varvec{w}}}_{1},\cdots ,{{\varvec{w}}}_{K}\right]\in {\mathbb{C}}^{M\times K}$. This optimization problem ${P}_{1}$ can be formulated as:

14a $${P}_{1}: {max}_{{\varvec{W}},\overline{{\varvec{\theta}}}} {R }_{sum}\left({\varvec{W}},\overline{{\varvec{\theta}} }\right)=\sum_{k=1}^{K}\text{log}\left(1+{\gamma }_{k}\right)$$

14b $$\text{s}.\text{t}. \sum_{k=1}^{K}{\Vert {{\varvec{w}}}_{k}\Vert }^{2}\le P,$$

14c $$\left|{\overline{\theta }}_{n}\right|=1, \forall n=1,\cdots ,N.$$

Based on the formulation of the optimization problem in ${P}_{1}$, both the objective function and constraints are non-convex, making it challenging to deal with such non-convex optimization problems. However, the proposed Fixed-Point (FP) algorithm can effectively handle the optimization problem presented in this paper. Additionally, we have also designed a low-complexity version of the FP method. The next section will provide a detailed description of the joint beamforming optimization algorithm using FP and low-complexity FP.

## Design of traditional beamforming optimization algorithms

In this section, we aim to optimize ${\varvec{W}}$ and $\overline{{\varvec{\theta}} }$ to maximize the system sum rate. Upon observation, the objective function in ${P}_{1}$ represents a typical fixed-point (FP) problem. First, we introduce an auxiliary variable to transform the objective function into a more manageable polynomial sum form in ${P}_{2}$. Secondly, by employing quadratic transformation, we convert the polynomial into a form (${P}_{3}$ and ${P}_{4}$) that allows optimization of the variables. Finally, we employ the methods of Lagrange multipliers and ADMM to respectively obtain closed-form solutions for optimizing ${\varvec{W}}$ and $\overline{{\varvec{\theta}} }$. The closed-form solutions obtained using these two approaches result in higher computational complexity. Therefore, we propose a low-complexity algorithm design as well.

First, introduce the auxiliary variable ${\eta }_{k}$^6,30^.Then, seeking the maximum value of $\text{log}\left(1+{\eta }_{k}\right)-{\eta }_{k}+\frac{\left(1+{\eta }_{k}\right){\gamma }_{k}}{1+{\gamma }_{k}}\text{log}\left(1+{\gamma }_{k}\right)$ with respect to ${\eta }_{k}$, $\text{log}\left(1+{\gamma }_{k}\right)$ can be expressed as:

15 $$\text{log}\left(1+{\gamma }_{k}\right)={max}_{{\eta }_{k\ge 0}}\text{ log}\left(1+{\eta }_{k}\right)-{\eta }_{k}+\frac{\left(1+{\eta }_{k}\right){\gamma }_{k}}{1+{\gamma }_{k}}.$$

Then, substituting ${\gamma }_{k}=\frac{{\left|{{\varvec{H}}}_{k}^{H}{{\varvec{w}}}_{k}\right|}^{2}}{\sum_{\text{j}=1,\text{j}\ne \text{k}}^{\text{K}}{\left|{{\varvec{H}}}_{k}^{H}{{\varvec{w}}}_{j}\right|}^{2}+{\upsigma }_{0}^{2}}$ into Eq. (15), we obtain:

16 $$\begin{aligned}   \text{log}\left(1+{\gamma }_{k}\right)&={max}_{{\eta }_{k\ge 0}}\text{ log}\left(1+{\eta }_{k}\right)-{\eta }_{k}+\frac{\left(1+{\eta }_{k}\right)\frac{{\left|{{\varvec{H}}}_{k}^{H}{{\varvec{w}}}_{\text{k}}\right|}^{2}}{\sum_{j=1,j\ne k}^{K}{\left|{{\varvec{H}}}_{k}^{H}{\mathbf{w}}_{j}\right|}^{2}+{\sigma }_{0}^{2}}}{1+\frac{{\left|{{\varvec{H}}}_{k}^{H}{{\varvec{w}}}_{\text{k}}\right|}^{2}}{\sum_{j=1,j\ne k}^{K}{\left|{{\varvec{H}}}_{k}^{H}{w}_{j}\right|}^{2}+{\sigma }_{0}^{2}}}\\ &={max}_{{\eta }_{k\ge 0}}\text{ log}\left(1+{\eta }_{k}\right)-{\eta }_{k}+\frac{\left(1+{\eta }_{k}\right)\frac{{\left|{{\varvec{H}}}_{k}^{H}{{\varvec{w}}}_{\text{k}}\right|}^{2}}{\sum_{j=1,j\ne k}^{K}{\left|{{\varvec{H}}}_{k}^{H}{{\varvec{w}}}_{j}\right|}^{2}+{\sigma }_{0}^{2}}}{\frac{\sum_{j=1,j\ne k}^{K}{\left|{{\varvec{H}}}_{k}^{H}{{\varvec{w}}}_{j}\right|}^{2}+{\sigma }_{0}^{2}}{\sum_{j=1,j\ne k}^{K}{\left|{{\varvec{H}}}_{k}^{H}{{\varvec{w}}}_{j}\right|}^{2}+{\sigma }_{0}^{2}}+\frac{{\left|{{\varvec{H}}}_{k}^{H}{{\varvec{w}}}_{\text{k}}\right|}^{2}}{\sum_{j=1,j\ne k}^{K}{\left|{{\varvec{H}}}_{k}^{H}{{\varvec{w}}}_{j}\right|}^{2}+{\sigma }_{0}^{2}}}\\ &={max}_{{\eta }_{k\ge 0}}\text{ log}\left(1+{\eta }_{k}\right)-{\eta }_{k}+\frac{\left(1+{\eta }_{k}\right)\frac{{\left|{{\varvec{H}}}_{k}^{H}{{\varvec{w}}}_{\text{k}}\right|}^{2}}{\sum_{j=1,j\ne k}^{K}{\left|{H}_{k}^{H}{{\varvec{w}}}_{j}\right|}^{2}+{\sigma }_{0}^{2}}}{\frac{\sum_{j=1}^{K}{\left|{{\varvec{H}}}_{k}^{H}{{\varvec{w}}}_{j}\right|}^{2}+{\sigma }_{0}^{2}}{\sum_{j=1,j\ne k}^{K}{\left|{{\varvec{H}}}_{k}^{H}{{\varvec{w}}}_{j}\right|}^{2}+{\sigma }_{0}^{2}}}\\ &={max}_{{\eta }_{k\ge 0}}\text{ log}\left(1+{\eta }_{k}\right)-{\eta }_{k}+\left(1+{\eta }_{k}\right)\frac{{\left|{{\varvec{H}}}_{k}^{H}{{\varvec{w}}}_{\text{k}}\right|}^{2}}{\sum_{j=1,j\ne k}^{K}{\left|{{\varvec{H}}}_{k}^{H}{{\varvec{w}}}_{j}\right|}^{2}+{\sigma }_{0}^{2}}\times \frac{\sum_{j=1,j\ne k}^{K}{\left|{{\varvec{H}}}_{k}^{H}{{\varvec{w}}}_{j}\right|}^{2}+{\sigma }_{0}^{2}}{\sum_{j=1}^{K}{\left|{{\varvec{H}}}_{k}^{H}{{\varvec{w}}}_{j}\right|}^{2}+{\sigma }_{0}^{2}}\\ &={max}_{{\eta }_{k\ge 0}}\text{ log}\left(1+{\eta }_{k}\right)-{\eta }_{k}+\left(1+{\eta }_{k}\right)\frac{{\left|{{\varvec{H}}}_{k}^{H}{{\varvec{w}}}_{\text{k}}\right|}^{2}}{\sum_{j=1}^{K}{\left|{{\varvec{H}}}_{k}^{H}{{\varvec{w}}}_{j}\right|}^{2}+{\sigma }_{0}^{2}}\end{aligned}.$$

Thus, substituting Eq. (16) into the objective function, the transformation into optimization problem ${P}_{2}$ can be expressed as follows:

17a $${P}_{2}: {max}_{{\varvec{W}},\overline{{\varvec{\theta}} },{\varvec{\eta}}} {F}_{1}\left({\varvec{W}},\overline{{\varvec{\theta}} },{\varvec{\eta}}\right)$$

17b $$\text{s}.\text{t}. \sum_{k=1}^{K}{\Vert {{\varvec{w}}}_{k}\Vert }^{2}\le P,$$

17c $$\left|{\overline{\theta }}_{n}\right|=1, \forall n=1,\cdots ,N.$$

17d $${\eta }_{k}\ge 0, k=1,\cdots ,K,$$

where $${F}_{1}\left({\varvec{W}},\overline{{\varvec{\theta}} },{\varvec{\eta}}\right)=\sum_{k=1}^{K}\left(\text{log}\left(1+{\eta }_{k}\right)-{\eta }_{k}+\left(1+{\eta }_{k}\right)\frac{{\left|{{\varvec{H}}}_{k}^{H}{{\varvec{w}}}_{\text{k}}\right|}^{2}}{\sum_{j=1}^{K}{\left|{{\varvec{H}}}_{k}^{H}{{\varvec{w}}}_{j}\right|}^{2}+{\sigma }_{0}^{2}}\right)$$, $${\varvec{\eta}}={\left[{\eta }_{1},\cdots ,{\eta }_{K}\right]}^{T}$$.

1. Fixing $${\varvec{W}},\overline{{\varvec{\theta}} }$$, solving for $${\varvec{\eta}}$$
  To obtain the optimized $${\varvec{W}}$$, we first need to calculate the optimal value of the introduced auxiliary variable $${\eta }_{k}$$. Upon observing the objective function, it is apparent that $${\varvec{\eta}}$$ represents a logarithmic linear function. Therefore, we need to take the first-order partial derivative of the objective function $${F}_{1}\left(W,\overline{\theta },{\varvec{\eta}}\right)$$ with respect to $${\eta }_{k}$$ and set the derivative equal to zero, leading to:
  18 $$\begin{aligned}   \frac{\partial {F}_{1}\left({\varvec{W}},\overline{{\varvec{\theta}} },{\varvec{\eta}}\right)}{{\eta }_{k}}&  =  \frac{1}{1+{\eta }_{k}}-1+\frac{{\left|{{\varvec{H}}}_{k}^{H}{{\varvec{w}}}_{\text{k}}\right|}^{2}}{\sum_{j=1}^{K}{\left|{{\varvec{H}}}_{k}^{H}{{\varvec{w}}}_{j}\right|}^{2}+{\sigma }_{0}^{2}}\\ &  = \frac{-{\eta }_{k}}{1+{\eta }_{k}}+\frac{{\left|{{\varvec{H}}}_{k}^{H}{{\varvec{w}}}_{\text{k}}\right|}^{2}}{\sum_{j=1}^{K}{\left|{{\varvec{H}}}_{k}^{H}{{\varvec{w}}}_{j}\right|}^{2}+{\sigma }_{0}^{2}}\end{aligned}  
.$$
  By setting $$\frac{-{\eta }_{k}}{1+{\eta }_{k}}+\frac{{\left|{{\varvec{H}}}_{k}^{H}{{\varvec{w}}}_{k}\right|}^{2}}{\sum_{j=1}^{K}{\left|{{\varvec{H}}}_{k}^{H}{{\varvec{w}}}_{j}\right|}^{2}+{\sigma }_{0}^{2}}$$ equal to 0, we obtain:
  19 $$\begin{aligned}   &\frac{{\left|{{\varvec{H}}}_{k}^{H}{{\varvec{w}}}_{k}\right|}^{2}}{\sum_{j=1}^{K}{\left|{{\varvec{H}}}_{k}^{H}{{\varvec{w}}}_{j}\right|}^{2}+{\sigma }_{0}^{2}}=\frac{{\eta }_{k}}{1+{\eta }_{k}}\\ &{\left|{{\varvec{H}}}_{k}^{H}{{\varvec{w}}}_{\text{k}}\right|}^{2}\left(1+{\eta }_{k}\right)=\left(\sum_{j=1}^{K}{\left|{{\varvec{H}}}_{k}^{H}{{\varvec{w}}}_{j}\right|}^{2}+{\sigma }_{0}^{2}\right){\eta }_{k}\\ &{\left|{{\varvec{H}}}_{k}^{H}{{\varvec{w}}}_{\text{k}}\right|}^{2}=\left(\sum_{j=1}^{K}{\left|{{\varvec{H}}}_{k}^{H}{{\varvec{w}}}_{j}\right|}^{2}+{\sigma }_{0}^{2}\right){\eta }_{k}-{\left|{{\varvec{H}}}_{k}^{H}{{\varvec{w}}}_{k}\right|}^{2}{\eta }_{k}\\ &{\eta }_{k}=\frac{{\left|{{\varvec{H}}}_{k}^{H}{{\varvec{w}}}_{k}\right|}^{2}}{\left(\sum_{j=1}^{K}{\left|{{\varvec{H}}}_{k}^{H}{{\varvec{w}}}_{j}\right|}^{2}+{\sigma }_{0}^{2}\right)-{\left|{{\varvec{H}}}_{k}^{H}{{\varvec{w}}}_{k}\right|}^{2}}\\ &{\eta }_{k}^{*}={\gamma }_{k}\end{aligned}  
,$$
  where $${\eta }_{k}^{*}$$ represents the optimal solution for $${\eta }_{k}$$. Substituting $${\eta }_{k}^{*}$$ into $${F}_{1}\left({\varvec{W}},\overline{{\varvec{\theta}} },{\varvec{\eta}}\right)$$, we can obtain:
  20 $$\begin{aligned}  {F}_{1}\left({\varvec{W}},\overline{{\varvec{\theta}} },{{\varvec{\eta}}}^{*}\right)&=\sum_{k=1}^{K}\left(\text{log}\left(1+{\eta }_{k}^{*}\right)-{\eta }_{k}^{*}+\left(1+{\eta }_{k}^{*}\right)\frac{{\left|{{\varvec{H}}}_{k}^{H}{{\varvec{w}}}_{k}\right|}^{2}}{\sum_{j=1}^{K}{\left|{{\varvec{H}}}_{k}^{H}{{\varvec{w}}}_{j}\right|}^{2}+{\sigma }_{0}^{2}}\right)\\ &={F}_{2}\left({\varvec{W}},\overline{{\varvec{\theta}} }\right) \end{aligned}  ,$$
  where $${{\varvec{\eta}}}^{*}={\left[{\eta }_{1}^{*},\cdots ,{\eta }_{K}^{*}\right]}^{T}$$. Therefore, from Eq. (20), it can be observed that there are only two optimization variables remaining: $${\varvec{W}}$$ and $$\overline{{\varvec{\theta}} }$$.
2. Fixing $$\overline{{\varvec{\theta}}}\text{ and}{{\varvec{\eta}} }^{*}$$, solving for $${\varvec{W}}$$
  Since the first two terms in $${F}_{2}\left({\varvec{W}},\overline{{\varvec{\theta}} }\right)$$ are not related to $${\varvec{W}}$$, and the third term with a fractional form is also non-convex, we need to perform quadratic transformation on $$\left(1+{\eta }_{k}^{*}\right)\frac{{\left|{{\varvec{H}}}_{k}^{H}{{\varvec{w}}}_{k}\right|}^{2}}{\sum_{j=1}^{K}{\left|{{\varvec{H}}}_{k}^{H}{{\varvec{w}}}_{j}\right|}^{2}+{\sigma }_{0}^{2}}$$ to express it as a sum of polynomials. Introducing an auxiliary variable $${\mu }_{k}$$, we can transform it into:
  21 $$\begin{aligned}   \sum_{k=1}^{K}\left(\left(1+{\eta }_{k}^{*}\right)\frac{{\left|{{\varvec{H}}}_{k}^{H}{{\varvec{w}}}_{k}\right|}^{2}}{\sum_{j=1}^{K}{\left|{{\varvec{H}}}_{k}^{H}{{\varvec{w}}}_{j}\right|}^{2}+{\sigma }_{0}^{2}}\right)&=\sum_{k=1}^{K}2\sqrt{{\widetilde{\eta }}_{k}^{*}}\mathfrak{R}\left\{{\mu }_{k}^{H}{{\varvec{H}}}_{k}^{H}{{\varvec{w}}}_{k}\right\}-\sum_{k=1}^{K}{\left|{\mu }_{k}\right|}^{2}\left(\sum_{j=1}^{K}{\left|{{\varvec{H}}}_{k}^{H}{{\varvec{w}}}_{j}\right|}^{2}+{\sigma }_{0}^{2}\right)\\ &={F}_{3}\left({\varvec{W}},{\varvec{\mu}}\right)\end{aligned}   .$$
  Since $$\overline{{\varvec{\theta}} }$$ and $${{\varvec{\eta}}}^{*}$$ can be treated as constants when optimizing the variable $${\varvec{W}}$$, the optimization problem of $${P}_{2}$$ can be transformed into $${P}_{3}$$:
  22a $${P}_{3}: {max}_{W,\overline{\theta },{\varvec{\eta}}} {F}_{3}\left({\varvec{W}},{\varvec{\mu}}\right)$$
  22b $$\text{s}.\text{t}. \sum_{k=1}^{K}{\Vert {{\varvec{w}}}_{k}\Vert }^{2}\le P,$$
  where $${\varvec{\mu}}={\left[{\mu }_{1},\cdots ,{\mu }_{K}\right]}^{T}$$. In order to solve the optimization problem for $${P}_{3}$$, we can utilize the method of Lagrange multipliers to transform this optimization problem into:
  23 $$\begin{aligned} {P}_{3}^{L}({\varvec{W}},\uplambda ,{\varvec{\mu}})&=\sum_{k=1}^{K}2\sqrt{{\widetilde{\eta }}_{k}^{*}}\mathfrak{R}\left\{{\mu }_{k}^{H}{{\varvec{H}}}_{k}^{H}{{\varvec{w}}}_{k}\right\}-\sum_{k=1}^{K}{\left|{\mu }_{k}\right|}^{2}\left(\sum_{j=1}^{K}{\left|{{\varvec{H}}}_{k}^{H}{{\varvec{w}}}_{j}\right|}^{2}+{\sigma }_{0}^{2}\right)\\ & \quad +\uplambda \left(\sum_{k=1}^{K}{{\varvec{w}}}_{k}^{H}{{\varvec{w}}}_{k}-P\right)\end{aligned} ,$$
  where $${P}_{3}^{L}({\varvec{W}},\uplambda ,{\varvec{\mu}})$$ represents the augmented Lagrangian function for the optimization problem $${P}_{3}$$, and $$\uplambda $$ represents the dual variable. Employing the train of thought of alternating optimization, we sequentially differentiate the three variables $${\varvec{W}},\uplambda $$ and $${\varvec{\mu}}$$ from Eq. (23), ultimately yielding their respective optimal values. First, we differentiate $${\mu }_{k}$$ to obtain the first-order partial derivative:
  24 $$\frac{\partial {P}_{3}^{L}({\varvec{\mu}},{\varvec{W}},\uplambda )}{{\mu }_{k}}= 2\sqrt{{\widetilde{\eta }}_{k}^{*}}{{\varvec{H}}}_{k}^{H}{{\varvec{w}}}_{k}-2{\mu }_{k}\left(\sum_{j=1}^{K}{\left|{{\varvec{H}}}_{k}^{H}{{\varvec{w}}}_{j}\right|}^{2}+{\sigma }_{0}^{2}\right).$$

Setting the first-order derivative of Eq. (24) equal to 0, we have:

25 $${\mu }_{k}^{*}=\frac{\sqrt{{\widetilde{\eta }}_{k}^{*}}{{\varvec{H}}}_{k}^{H}{{\varvec{w}}}_{\text{k}}}{\sum_{j=1}^{K}{\left|{{\varvec{H}}}_{k}^{H}{{\varvec{w}}}_{j}\right|}^{2}+{\sigma }_{0}^{2}},$$

where ${\mu }_{k}^{*}$ represents the optimal solution for the auxiliary variable ${\mu }_{k}$. Substituting ${\mu }_{k}^{*}$ into ${P}_{3}^{L}({\varvec{\mu}},W,\uplambda )$, we obtain ${P}_{3}^{L}({{\varvec{\mu}}}^{*},{\varvec{W}},\uplambda )$, where ${{\varvec{\mu}}}^{*}={\left[{\mu }_{1}^{*},\cdots ,{\mu }_{K}^{*}\right]}^{T}$. Then, the first derivative function of $\uplambda$ is solved for ${P}_{3}^{L}({{\varvec{\mu}}}^{*},{\varvec{W}},\uplambda )$, and we can get:

26 $$\frac{\partial {P}_{3}^{L}({{\varvec{\mu}}}^{*},{\varvec{W}},\uplambda )}{\uplambda }=\sum_{k=1}^{K}{{\varvec{w}}}_{k}^{H}{{\varvec{w}}}_{k}-P.$$

As $\uplambda >0$ and ${{\varvec{w}}}_{k}^{H}{{\varvec{w}}}_{k}-P\le 0$, the function ${P}_{3}^{L}({{\varvec{\mu}}}^{*},{\varvec{W}},\uplambda )$ with respect to $\uplambda$ is a monotonically decreasing function. Therefore, when ${{\varvec{w}}}_{k}^{H}{{\varvec{w}}}_{k}=P$, the function ${P}_{3}^{L}({{\varvec{\mu}}}^{*},{\varvec{W}},\uplambda )$ attains its maximum value. If the value of ${{{\varvec{w}}}_{k}^{*}}^{H}{{\varvec{w}}}_{k}^{*}<P$, where ${{\varvec{w}}}_{k}^{*}$ represents the optimal beamforming vector, then the minimum value of $\uplambda$ should be taken to achieve the maximum value of the function ${P}_{3}^{L}({{\varvec{\mu}}}^{*},{\varvec{W}},\uplambda )$. Thus, the optimal solution of $\uplambda$, denoted as ${\uplambda }^{*}$, can be expressed as:

27 $${\uplambda }^{*}=min\left\{{\uplambda }^{*}\ge 0:\sum_{k=1}^{K}{\Vert {{\varvec{w}}}_{k}^{*}\Vert }^{2}\le P\right\}.$$

Substituting the optimal solution ${\uplambda }^{*}$ for $\uplambda$ into the function ${P}_{3}^{L}({{\varvec{\mu}}}^{*},{\varvec{W}},\uplambda )$ to obtain ${P}_{3}^{L}({{\varvec{\mu}}}^{*},{\varvec{W}},{\uplambda }^{*})$, then differentiating ${w}_{k}$, we get:

28 $$\frac{\partial {P}_{3}^{L}({{\varvec{\mu}}}^{*},W,{\uplambda }^{*})}{{{\varvec{w}}}_{k}}=2\sqrt{{\widetilde{\eta }}_{k}^{*}}{\mu }_{k}^{*}{{\varvec{H}}}_{k}-2\sum_{j=1}^{K}{\left|{\mu }_{j}^{*}\right|}^{2}\sum_{k=1}^{K}{{\varvec{H}}}_{j}{{\varvec{H}}}_{j}^{H}{{\varvec{w}}}_{k}-2{\uplambda }^{*}{{\varvec{I}}}_{M}{{\varvec{w}}}_{k}.$$

Setting the first-order derivative equal to zero, we have:

29 $${{\varvec{w}}}_{k}^{*}=\sqrt{{\widetilde{\eta }}_{k}^{*}}{\mu }_{k}^{*}{\left(\sum_{j=1}^{K}{\left|{\mu }_{j}^{*}\right|}^{2}{{\varvec{H}}}_{j}{{\varvec{H}}}_{j}^{H}+{\uplambda }^{*}{{\varvec{I}}}_{N}\right)}^{-1}{{\varvec{H}}}_{k}.$$

Therefore, ${{\varvec{W}}}^{*}={\left[{{\varvec{w}}}_{1}^{*},\cdots ,{{\varvec{w}}}_{K}^{*}\right]}^{T}$ yields the optimized beamforming matrix.In addition, the calculation of ${{\varvec{w}}}_{k}^{*}$ involves matrix inversion, which leads to a high computational complexity.

As ${{\varvec{H}}}_{k}^{H}=\left({{\varvec{h}}}_{BS,k}^{H}+{\overline{{\varvec{\theta}}} }^{H}{{\varvec{h}}}_{a}\right)$, ${F}_{2}\left({\varvec{W}},\overline{{\varvec{\theta}} }\right)$ can be written as:

30 $${F}_{2}\left({\varvec{W}},\overline{{\varvec{\theta}} }\right)=\sum_{k=1}^{K}\left(\text{log}\left(1+{\eta }_{k}^{*}\right)-{\eta }_{k}^{*}+\left(1+{\eta }_{k}^{*}\right)\frac{{\left|\left({{\varvec{h}}}_{BS,k}^{H}+{\overline{{\varvec{\theta}}} }^{H}{{\varvec{h}}}_{a}\right){{\varvec{w}}}_{k}\right|}^{2}}{\sum_{j=1}^{K}{\left|\left({{\varvec{h}}}_{BS,k}^{H}+{\overline{{\varvec{\theta}}} }^{H}{{\varvec{h}}}_{a}\right){{\varvec{w}}}_{j}\right|}^{2}+{\sigma }_{0}^{2}}\right).$$

Observing formula (30), $\overline{{\varvec{\theta}} }$ is only related to $\left(1+{\eta }_{k}^{*}\right)\frac{{\left|\left({{\varvec{h}}}_{BS,k}^{H}+{\overline{{\varvec{\theta}}} }^{H}{{\varvec{h}}}_{a}\right){{\varvec{w}}}_{k}\right|}^{2}}{\sum_{j=1}^{K}{\left|\left({{\varvec{h}}}_{BS,k}^{H}+{\overline{{\varvec{\theta}}} }^{H}{{\varvec{h}}}_{a}\right){{\varvec{w}}}_{j}\right|}^{2}+{\sigma }_{0}^{2}}$, so it is only necessary to transform this term and then optimize to obtain the optimal solution. By introducing an auxiliary variable ${\xi }_{k}$ and using quadratic transformation, we can obtain:

31 $$ \begin{aligned} & \sum_{k=1}^{K}\left(\left(1+{\eta }_{k}^{*}\right)\frac{{\left|\left({{\varvec{h}}}_{BS,k}^{H}+{\overline{{\varvec{\theta}}} }^{H}{{\varvec{h}}}_{a}\right){{\varvec{w}}}_{k}\right|}^{2}}{\sum_{j=1}^{K}{\left|\left({{\varvec{h}}}_{BS,k}^{H}+{\overline{{\varvec{\theta}}} }^{H}{{\varvec{h}}}_{a}\right){{\varvec{w}}}_{j}\right|}^{2}+{\sigma }_{0}^{2}}\right)\\&\quad=\sum_{k=1}^{K}2\sqrt{{\widetilde{\eta }}_{k}^{*}}\mathfrak{R}\left\{{\xi }_{k}^{H}\left({{\varvec{h}}}_{BS,k}^{H}+{\overline{{\varvec{\theta}}} }^{H}{{\varvec{h}}}_{a}\right){{\varvec{w}}}_{k}\right\}-\sum_{k=1}^{K}{\left|{\xi }_{k}\right|}^{2}\left(\sum_{j=1}^{K}{\left|\left({{\varvec{h}}}_{BS,k}^{H}+{\overline{{\varvec{\theta}}} }^{H}{{\varvec{h}}}_{a}\right){{\varvec{w}}}_{j}\right|}^{2}+{\sigma }_{0}^{2}\right)\\&\quad={F}_{4}\left(\overline{{\varvec{\theta}} },{\varvec{\xi}}\right)\end{aligned},$$

where ${\varvec{\xi}}={\left[{\xi }_{1},\cdots ,{\xi }_{K}\right]}^{T}$, differentiating ${\xi }_{k}$ with respect to the relevant variables yields the first-order partial derivative:

32 $$ \begin{aligned}{}&\frac{\partial \left({F}_{4}\left(\overline{\theta },{\varvec{\xi}}\right)\right)}{{\xi }_{k}}\\ & \quad=2\sqrt{{\widetilde{\eta }}_{k}^{*}}\left({{\varvec{h}}}_{BS,k}^{H}+{\overline{{\varvec{\theta}}} }^{H}{{\varvec{h}}}_{a}\right){{\varvec{w}}}_{k}-2{\xi }_{k}\left(\sum_{j=1}^{K}{\left|\left({{\varvec{h}}}_{BS,k}^{H}+{\overline{{\varvec{\theta}}} }^{H}{{\varvec{h}}}_{a}\right){{\varvec{w}}}_{j}\right|}^{2}+{\sigma }_{0}^{2}\right)\end{aligned} .$$

here, setting Eq. (32) equal to zero, we have:

33 $${\xi }_{k}^{*}=\frac{\sqrt{{\widetilde{\eta }}_{k}^{*}}\left({{\varvec{h}}}_{BS,k}^{H}+{\overline{{\varvec{\theta}}} }^{H}{{\varvec{h}}}_{a}\right){{\varvec{w}}}_{k}}{\sum_{j=1}^{K}{\left|\left({{\varvec{h}}}_{BS,k}^{H}+{\overline{{\varvec{\theta}}} }^{H}{{\varvec{h}}}_{a}\right){{\varvec{w}}}_{j}\right|}^{2}+{\sigma }_{0}^{2}}.$$

Therefore, ${{\varvec{\xi}}}^{\boldsymbol{*}}={\left[{\xi }_{1}^{*},\cdots ,{\xi }_{K}^{*}\right]}^{{\varvec{T}}}$. By substituting ${{\varvec{\xi}}}^{\boldsymbol{*}}$ into ${F}_{4}\left(\overline{{\varvec{\theta}} },{\varvec{\xi}}\right)$, we obtain ${F}_{4}\left(\overline{{\varvec{\theta}} },{{\varvec{\xi}}}^{\boldsymbol{*}}\right)$, and the optimization only needs to be performed on $\overline{{\varvec{\theta}} }$. Furthermore, ${F}_{4}\left(\overline{{\varvec{\theta}} },{{\varvec{\xi}}}^{\boldsymbol{*}}\right)$ can be rewritten as:

34 $$\begin{aligned}{F}_{4}\left(\overline{{\varvec{\theta}} },{{\varvec{\xi}}}^{\boldsymbol{*}}\right)&=\sum_{k=1}^{K}2\sqrt{{\widetilde{\eta }}_{k}^{*}}\mathfrak{R}\left\{{\xi }_{k}^{*}\left({{\varvec{h}}}_{BS,k}^{H}+{\overline{{\varvec{\theta}}} }^{H}{{\varvec{h}}}_{a}\right){{\varvec{w}}}_{k}\right\}-\sum_{k=1}^{K}{\left|{\xi }_{k}^{*}\right|}^{2}\left(\sum_{j=1}^{K}{\left|\left({{\varvec{h}}}_{BS,k}^{H}+{\overline{{\varvec{\theta}}} }^{H}{{\varvec{h}}}_{a}\right){{\varvec{w}}}_{j}\right|}^{2}+{\sigma }_{0}^{2}\right)\\ &=\sum_{k=1}^{K}2\sqrt{{\widetilde{\eta }}_{k}^{*}}\mathfrak{R}\left\{{\left({\xi }_{k}^{*}\right)}^{H}\left({{\varvec{h}}}_{BS,k}^{H}+{\overline{{\varvec{\theta}}} }^{H}{{\varvec{h}}}_{a}\right){{\varvec{w}}}_{k}\right\}\\ & \quad-\sum_{k=1}^{K}{\left({\xi }_{k}^{*}\right)}^{H}\left(\sum_{j=1}^{K}\left(\left({{\varvec{h}}}_{BS,k}^{H}+{\overline{{\varvec{\theta}}} }^{H}{{\varvec{h}}}_{a}\right){{\varvec{w}}}_{j}\right){\left(\left({{\varvec{h}}}_{BS,k}^{H}+{\overline{{\varvec{\theta}}} }^{H}{{\varvec{h}}}_{a}\right){{\varvec{w}}}_{j}\right)}^{H}+{\sigma }_{0}^{2}\right){\xi }_{k}^{*}\end{aligned}  
.$$

here, expanding ${\left({\xi }_{k}^{*}\right)}^{H}\left(\left({{\varvec{h}}}_{BS,k}^{H}+{\overline{{\varvec{\theta}}} }^{H}{{\varvec{h}}}_{a}\right){{\varvec{w}}}_{j}\right)$ can be expressed as:

35 $$\begin{aligned}{\left({\xi }_{k}^{*}\right)}^{H}\left(\left({{\varvec{h}}}_{BS,k}^{H}+{\overline{{\varvec{\theta}}} }^{H}{{\varvec{h}}}_{a}\right){{\varvec{w}}}_{j}\right)&={\left({\xi }_{k}^{*}\right)}^{H}{{\varvec{h}}}_{BS,k}^{H}{{\varvec{w}}}_{j}+{\left({\xi }_{k}^{*}\right)}^{H}{\overline{{\varvec{\theta}}} }^{H}{{\varvec{h}}}_{a}{{\varvec{w}}}_{j}\\&={C}_{k,j}+{\overline{{\varvec{\theta}}} }^{H}{{\varvec{G}}}_{k,j}\end{aligned}  ,$$

where ${C}_{k,j}$ and ${G}_{k,j}$ are expressed as:

36a $${C}_{k,j}={\left({\xi }_{k}^{*}\right)}^{H}{{\varvec{h}}}_{BS,k}^{H}{{\varvec{w}}}_{j}.$$

36b $${{\varvec{G}}}_{k,j}={\left({\xi }_{k}^{*}\right)}^{H}{{\varvec{h}}}_{a}{{\varvec{w}}}_{j}.$$

Substituting Eq. (35) into Eq. (34), we obtain:

37 $$ \begin{aligned} {F}_{4}\left(\overline{{\varvec{\theta}} },{{\varvec{\xi}}}^{\boldsymbol{*}}\right)&=\sum_{k=1}^{K}2\sqrt{{\widetilde{\eta }}_{k}^{*}}\mathfrak{R}\left\{{C}_{k,k}+{\overline{{\varvec{\theta}}} }^{H}{{\varvec{G}}}_{k,k}\right\}-\sum_{k=1}^{K}\left(\sum_{j=1}^{\text{K}}\left({C}_{k,j}+{\overline{{\varvec{\theta}}} }^{H}{{\varvec{G}}}_{k,j}\right)\left({C}_{k,j}^{*}+{{\varvec{G}}}_{k,j}^{H}\overline{{\varvec{\theta}} }\right)+{\left({\xi }_{k}^{*}\right)}^{H}{\sigma }_{0}^{2}{\xi }_{k}^{*}\right)\\ &=\sum_{k=1}^{K}2\sqrt{{\widetilde{\eta }}_{k}^{*}}\mathfrak{R}\left\{{\overline{{\varvec{\theta}}} }^{H}{G}_{k,k}\right\}+\sum_{k=1}^{K}2\sqrt{{\widetilde{\eta }}_{k}^{*}}\mathfrak{R}\left\{{C}_{k,k}\right\}\\ & \quad-\sum_{k=1}^{K}\left(\sum_{j=1}^{\text{K}}\left({C}_{k,j}{C}_{k,j}^{*}+{C}_{k,j}{{\varvec{G}}}_{k,j}^{H}\overline{{\varvec{\theta}} }+{\overline{{\varvec{\theta}}} }^{H}{{\varvec{G}}}_{k,j}{C}_{k,j}^{*}+{\overline{{\varvec{\theta}}} }^{H}{{\varvec{G}}}_{k,j}{{\varvec{G}}}_{k,j}^{H}\overline{{\varvec{\theta}} }\right)+{\left({\xi }_{k}^{*1}\right)}^{H}{\sigma }_{0}^{2}{\xi }_{k}^{*1}\right)\\&=-\sum_{k=1}^{K}\sum_{j=1}^{K}{\overline{{\varvec{\theta}}} }^{H}{{\varvec{G}}}_{k,j}{{\varvec{G}}}_{k,j}^{H}\overline{{\varvec{\theta}} }+2\sum_{k=1}^{K}\sqrt{{\widetilde{\eta }}_{k}^{*}}\mathfrak{R}\left\{{\overline{{\varvec{\theta}}} }^{H}{{\varvec{G}}}_{k,k}\right\}-2\sum_{k=1}^{K}\sum_{j=1}^{K}\mathfrak{R}\left\{{\overline{{\varvec{\theta}}} }^{H}{C}_{k,j}^{*}{{\varvec{G}}}_{k,j}\right\}+\sum_{k=1}^{K}2\sqrt{{\widetilde{\eta }}_{k}^{*}}\mathfrak{R}\left\{{C}_{k,k}\right\}\\ & \quad-\sum_{k=1}^{K}\sum_{j=1}^{K}{C}_{k,j}{C}_{k,j}^{*}-\sum_{k=1}^{K}{\left({\xi }_{k}^{*1}\right)}^{H}{\sigma }_{0}^{2}{\xi }_{k}^{*1}\end{aligned} ,$$

here, $2\mathfrak{R}\left\{{\overline{{\varvec{\theta}}} }^{H}{C}_{k,j}^{*}{{\varvec{G}}}_{k,j}\right\}={\overline{{\varvec{\theta}}} }^{H}{{\varvec{G}}}_{k,j}{C}_{k,j}^{*}+{C}_{k,j}{{\varvec{G}}}_{k,j}^{H}\overline{{\varvec{\theta}} }$. Therefore, ${F}_{4}\left(\overline{{\varvec{\theta}} },{{\varvec{\xi}}}^{\boldsymbol{*}}\right)$ can be rewritten as:

38 $${F}_{4}\left(\overline{{\varvec{\theta}} },{{\varvec{\xi}}}^{\boldsymbol{*}}\right)=-{\overline{{\varvec{\theta}}} }^{H}{\varvec{U}}\overline{{\varvec{\theta}} }+2\mathfrak{R}\left\{{\overline{{\varvec{\theta}}} }^{H}{\varvec{V}}\right\}+\kappa ,$$

among them:

39a $${\varvec{U}}=\sum_{k=1}^{K}\sum_{j=1}^{K}{{\varvec{G}}}_{k,j}{{\varvec{G}}}_{k,j}^{H}.$$

39b $${\varvec{V}}=\sum_{k=1}^{K}\sqrt{{\widetilde{\eta }}_{k}^{*}}{{\varvec{G}}}_{k,k}-\sum_{k=1}^{K}\sum_{j=1}^{K}{C}_{k,j}^{*}{{\varvec{G}}}_{k,j}.$$

39c $$\kappa =\sum_{k=1}^{K}2\sqrt{{\widetilde{\eta }}_{k}^{*}}\mathfrak{R}\left\{{C}_{k,k}\right\}-\sum_{k=1}^{K}\sum_{j=1}^{K}{C}_{k,j}{C}_{k,j}^{*}-\sum_{\text{k}=1}^{\text{K}}{\left({\xi }_{k}^{*}\right)}^{H}{\sigma }_{0}^{2}{\xi }_{k}^{*}.$$

Therefore, the problem of optimizing $\overline{{\varvec{\theta}} }$ can be formulated as ${P}_{4}$:

40a $${P}_{4}: {max}_{\overline{{\varvec{\theta}}}} {F }_{4}\left(\overline{{\varvec{\theta}} },{{\varvec{\xi}}}^{\boldsymbol{*}}\right)$$

40b $$\text{s}.\text{t}. \left|{\overline{\theta }}_{n}\right|=1, \forall n=1,\cdots ,N.$$

It can be observed that ${P}_{4}$ is a Quadratically Constrained Quadratic Programming (QCQP) problem. Therefore, we can solve it using the Alternating Direction Method of Multipliers (ADMM) approach. First, we introduce an auxiliary vector $\mathbf{\varrho }$, and then the problem ${P}_{4}$ can be transformed into ${P}_{5}$:

41a $${P}_{5}: {max}_{\mathbf{\varrho },\overline{{\varvec{\theta}}}} {F }_{4}\left(\mathbf{\varrho },{{\varvec{\xi}}}^{\boldsymbol{*}}\right)-\frac{\varpi }{2}{\Vert \mathbf{\varrho }-\overline{{\varvec{\theta}}}\Vert  }^{2}$$

41b $$\text{s}.\text{t}. \mathbf{\varrho }=\overline{{\varvec{\theta}} },$$

41c $$\left|{\overline{\theta }}_{n}\right|=1, \forall n=1,\cdots ,N.$$

where $\varpi >0$ is a penalty parameter. Then, introducing an auxiliary variable $\widetilde{{\varvec{\uplambda}}}$, the Lagrangian transformation of ${P}_{5}$ can be expressed as:

42 $$\begin{aligned}   \mathcal{L}(\overline{{\varvec{\theta}} },\mathbf{\varrho },\widetilde{{\varvec{\uplambda}}})&=-{\mathbf{\varrho }}^{H}{\varvec{U}}\mathbf{\varrho }+2\mathfrak{R}\left\{{\mathbf{\varrho }}^{H}{\varvec{V}}\right\}+\kappa -\frac{\varpi }{2}{\Vert \mathbf{\varrho }-\overline{{\varvec{\theta}}}\Vert  }^{2}+\mathfrak{R}\left\{{\widetilde{{\varvec{\uplambda}}}}^{H}\left(\mathbf{\varrho }-\overline{{\varvec{\theta}} }\right)\right\}\\&=-{\mathbf{\varrho }}^{H}{\varvec{U}}\mathbf{\varrho }-\frac{\varpi }{2}{\Vert \mathbf{\varrho }-\overline{{\varvec{\theta}}}\Vert  }^{2}+\mathfrak{R}\left\{2{\mathbf{\varrho }}^{H}{\varvec{V}}+{\widetilde{{\varvec{\uplambda}}}}^{H}\left(\mathbf{\varrho }-\overline{{\varvec{\theta}} }\right)\right\}\end{aligned}  
.$$

Then, based on the ADMM method to solve Eq. (42), we obtain:

43 $${\overline{{\varvec{\theta}}} }^{t+1}={argmax}_{\overline{\theta }} \mathcal{L}(\overline{{\varvec{\theta}} },{\mathbf{\varrho }}^{t},{\widetilde{{\varvec{\uplambda}}}}^{t}),$$

44 $${\mathbf{\varrho }}^{t+1}={argmax}_{\mathbf{\varrho }} \mathcal{L}({\overline{{\varvec{\theta}}} }^{t+1},\mathbf{\varrho },{\widetilde{{\varvec{\uplambda}}}}^{t}),$$

45 $${\widetilde{{\varvec{\uplambda}}}}^{t+1}={\widetilde{{\varvec{\uplambda}}}}^{t}-\varpi \left({\mathbf{\varrho }}^{t+1}-{\overline{{\varvec{\theta}}} }^{t+1}\right),$$

where $\widetilde{{\varvec{\uplambda}}}$ is a complex vector. *t* is the number of iterations. Differentiating $\mathcal{L}(\overline{{\varvec{\theta}} },{\mathbf{\varrho }}^{t},{\widetilde{{\varvec{\uplambda}}}}^{t})$ with respect to $\overline{{\varvec{\theta}} }$ yields the first-order partial derivative:

46 $$\frac{\delta \left(\mathcal{L}(\overline{{\varvec{\theta}} },{\mathbf{\varrho }}^{t},{\widetilde{{\varvec{\uplambda}}}}^{t})\right)}{\overline{{\varvec{\theta}}} }=\varpi \left({\mathbf{\varrho }}^{t}-\overline{{\varvec{\theta}} }\right)-{\widetilde{{\varvec{\uplambda}}}}^{t}.$$

Let Eq. (46) be equal to 0, then:

47 $${\overline{{\varvec{\theta}}} }^{t+1}={\mathbf{\varrho }}^{t}-\frac{1}{\varpi }{\widetilde{{\varvec{\uplambda}}}}^{t}.$$

Due to the constraints on the RIS reflection coefficients, $\left|{\widetilde{\theta }}_{n}\right|=1$, where $\forall n=1,\cdots ,{N}_{r}$, the optimal solution obtained from Eq. (47) can be expressed as the calculated angle:

48 $${\angle }
{\overline{\theta }}_{n}^{*}={\angle }{\widetilde{\overline{\theta }}}_{n}^{*},$$

where ${\widetilde{\overline{\theta }}}_{n}^{*}$ represents the $n$-th element obtained from the optimized closed-form solution of ${\overline{{\varvec{\theta}}} }^{*}$. Next, differentiating $\mathcal{L}({\overline{{\varvec{\theta}}} }^{t+1},\mathbf{\varrho },{\widetilde{{\varvec{\uplambda}}}}^{t})$ with respect to $\mathbf{\varrho }$ yields the first-order partial derivative:

49 $$\frac{\delta \left(\mathcal{L}({\overline{{\varvec{\theta}}} }^{t+1},\mathbf{\varrho },{\widetilde{{\varvec{\uplambda}}}}^{t})\right)}{\mathbf{\varrho }}=-2{\varvec{U}}\mathbf{\varrho }-\varpi \left(\mathbf{\varrho }-{\overline{{\varvec{\theta}}} }^{t+1}\right)+2{\varvec{V}}+{\widetilde{{\varvec{\uplambda}}}}^{t}.$$

Let Eq. (49) be equal to 0, then :

50 $${\mathbf{\varrho }}^{t+1}={\left(2{\varvec{U}}+\varpi {{\varvec{I}}}_{N}\right)}^{-1}\left(2{\varvec{V}}+{\widetilde{{\varvec{\uplambda}}}}^{t}+\varpi {\overline{{\varvec{\theta}}} }^{t+1}\right),$$

where, parameter $\varpi =\tau {\Vert {\varvec{U}}\Vert }_{2}$, $\tau \ge 1$. Finally, the optimal solution of ${\widetilde{{\varvec{\uplambda}}}}^{t+1}$ is obtained based on Formula (45) until the algorithm converges.Furthermore, it can be observed that the matrix inversion operation in $\mathbf{\varrho }$ leads to a high computational complexity. Additionally, we can see that in Eq. (47), the optimized closed-form solution ${\overline{{\varvec{\theta}}} }^{t+1}$ is obtained through linear operations, resulting in lower computational complexity. However, the ADMM algorithm requires iterative calculations of ${\mathbf{\varrho }}^{t+1}$ and ${\widetilde{{\varvec{\uplambda}}}}^{t+1}$ to obtain ${\overline{{\varvec{\theta}}} }^{t+1}$, and there is a matrix inversion process involved in obtaining ${\mathbf{\varrho }}^{t+1}$. Therefore, employing the ADMM algorithm to obtain the final closed-form solution for RIS passive beamforming optimization would introduce a higher computational complexity.

## Proposed low complexity algorithm design

In this section, Based on the drawbacks of traditional beamforming optimization algorithms discussed in the previous section, we propose a low-complexity optimization algorithm design based on the "Woodbury transformation" and "scalar transformation".

1. Closed-form solution based on the "Woodbury transformation" for ${\varvec{W}}$
  The closed-form solution for W obtained from Eq. (29) involves matrix inversion, which results in high computational complexity $\mathcal{O}\left({M}^{3}\right)$. Therefore, using the "Woodbury transformation" can reduce the computational complexity of calculating $\mathcal{O}\left({M}^{2}\right)$. First, we transform Eq. (29) into the form of the "Woodbury transformation". Let $\boldsymbol{\rm B}=\sum_{j=1}^{K}{\left|{\mu }_{j}^{*}\right|}^{2}{{\varvec{H}}}_{j}{{\varvec{H}}}_{j}^{H}$, ${x}_{j}={\left|{\mu }_{j}^{*}\right|}^{2}$, then B can be written as:
  51 $${\varvec{B}}=\left(\sqrt{{x}_{1}}\begin{array}{ccc}{{\varvec{H}}}_{1}& \begin{array}{ccc}\cdots & {\sqrt{{x}_{j}}{\varvec{H}}}_{j}& \cdots \end{array}& {\sqrt{{x}_{K}}{\varvec{H}}}_{K}\end{array}\right)\left(\begin{array}{c}{\left(\sqrt{{x}_{1}}{{\varvec{H}}}_{1}\right)}^{H}\\ \begin{array}{c}\vdots \\ {\left(\sqrt{{x}_{j}}{{\varvec{H}}}_{j}\right)}^{H}\\ \vdots \end{array}\\ {\left(\sqrt{{x}_{K}}{{\varvec{H}}}_{K}\right)}^{H}\end{array}\right),$$
  where, let $${\varvec{D}}=\left(\sqrt{{x}_{1}}\begin{array}{ccc}{{\varvec{H}}}_{1}& \begin{array}{ccc}\cdots & {\sqrt{{x}_{j}}{\varvec{H}}}_{j}& \cdots \end{array}& {\sqrt{{x}_{K}}{\varvec{H}}}_{K}\end{array}\right)\in {\mathbb{C}}^{M\times K}$$. Therefore, ${\varvec{B}}={\varvec{D}}{{\varvec{D}}}^{H}$. Let ${\varvec{E}}={{\varvec{D}}}^{H}\in {\mathbb{C}}^{K\times M}$, then ${\varvec{B}}$ can be written as:
  52 $${\varvec{B}}={{\varvec{E}}}^{H}{\varvec{E}}.$$
  Substituting ${\varvec{B}}={{\varvec{E}}}^{H}{\varvec{E}}$ into formula (29), we have:
  53 $${{\varvec{w}}}_{k}^{*}=\sqrt{{\widetilde{\eta }}_{k}^{*}}{\mu }_{k}^{*}{\left({{\varvec{E}}}^{H}{\varvec{E}}+{\uplambda }^{*}{{\varvec{I}}}_{N}\right)}^{-1}{{\varvec{H}}}_{k}.$$
  Therefore, Eq. (53) is transformed into the form based on the "Woodbury transformation". Hence, ${\left({{\varvec{E}}}^{H}{\varvec{E}}+{\uplambda }^{*}{{\varvec{I}}}_{N}\right)}^{-1}$ based on the "Woodbury transformation" can be written as:
  54 $${\left({{\varvec{E}}}^{H}{\varvec{E}}+{\uplambda }^{*}{{\varvec{I}}}_{N}\right)}^{-1}=\frac{1}{{\uplambda }^{*}}\left({{\varvec{I}}}_{M}-{{\varvec{E}}}^{H}{\left({\uplambda }^{*}{{\varvec{I}}}_{K}+{\varvec{E}}{{\varvec{E}}}^{H}\right)}^{-1}{\varvec{E}}\right).$$
  Based on the derivation in Eq. (54), formula (29) can be rewritten as:
  55 $${{\varvec{w}}}_{k}^{*}=\sqrt{{\widetilde{\eta }}_{k}^{*}}{\mu }_{k}^{*}\frac{1}{{\uplambda }^{*}}\left({{\varvec{I}}}_{M}-{{\varvec{E}}}^{H}{\left({\uplambda }^{*}{{\varvec{I}}}_{K}+{\varvec{E}}{{\varvec{E}}}^{H}\right)}^{-1}{\varvec{E}}\right){{\varvec{H}}}_{k},$$
  In the obtained optimized closed-form solution ${{\varvec{w}}}_{k}^{*}$, there is also a matrix inversion operation, denoted as ${\left({\uplambda }^{*}{{\varvec{I}}}_{K}+{\varvec{E}}{{\varvec{E}}}^{H}\right)}^{-1}$, but the computational complexity at this stage is ${K}^{3}$.where the number of base station antennas $M>K$, or when $M>>K$, after the transformation, the computational complexity of ${K}^{3}$ can be ignored, leaving only the computational complexity of matrix ${{\varvec{E}}}^{H}$ multiplied by ${\varvec{E}}$, denoted as $\mathcal{O}\left({M}^{2}\right)$.
2. Closed-form solution based on the "Scalar transformation" for $\overline{{\varvec{\theta}} }$
  From the objective function ${F}_{4}\left(\overline{{\varvec{\theta}} },{{\varvec{\xi}}}^{\boldsymbol{*}}\right)=-{\overline{{\varvec{\theta}}} }^{H}{\varvec{U}}\overline{{\varvec{\theta}} }+2\mathfrak{R}\left\{{\overline{{\varvec{\theta}}} }^{H}{\varvec{V}}\right\}+\kappa$ in ${P}_{4}$, we can observe that it is a quadratic function with respect to an element $\overline{{\varvec{\theta}} }$ in ${\overline{\theta }}_{n}$. It can be expressed as:
  56 $${F}_{4}({\overline{\theta }}_{n})=-{u}_{nn}{{\overline{\theta }}_{n}}^{2}+2\mathfrak{R}\left\{{{\overline{\theta }}_{n}}^{H}{V}_{n}\right\}-{\overline{\theta }}_{n}\left(\sum_{l=1, l\ne n}^{{N}_{r}}{\overline{\theta }}_{l}({u}_{ln}+{u}_{nl})\right)+C,$$
  where ${u}_{nn}$, ${V}_{n}$, ${u}_{ln}$, and ${u}_{nl}$ represent the $j$-th diagonal element of matrix $U$, the n-th element of vector ${V}_{n}$, the element in the *l*-th row and *n*-th column of matrix $U$, and the element in the *n*-th row and *l*-th column of matrix $U$, respectively. C represents a term independent of ${\overline{\theta }}_{n}$. Therefore, Eq. (61) represents the scalar quadratic function of the *n*-th element of the RIS reflection that we want to optimize. Taking the derivative of ${\overline{\theta }}_{n}$, we can obtain:
  57 $$\frac{\partial \left({F}_{4}({\overline{\theta }}_{n})\right)}{{\overline{\theta }}_{n}}=-2{u}_{nn}{\overline{\theta }}_{n}+2{V}_{n}-\sum_{l=1, l\ne n}^{{N}_{r}}{\overline{\theta }}_{l}({u}_{ln}+{u}_{nl}).$$
  Then let the formula (57) be equal to 0, we obtain:
  58 $${\overline{\theta }}_{n}=\frac{2{V}_{n}-\sum_{l=1, l\ne n}^{N}{\overline{\theta }}_{l}({u}_{ln}+{u}_{nl})}{2{u}_{nn}}.$$
  As the reflection coefficients of RIS need to satisfy the constraint in formula (13), we introduce an auxiliary vector ${\varvec{\updelta}}$. Substituting it into Eq. (58), we have:
  59 $${{\overline{\theta }}_{n}}^{*}=\frac{2{V}_{n}-\sum_{l=1, l\ne n}^{N}{\overline{\theta }}_{l}({u}_{ln}+{u}_{nl})}{2{\delta }_{n}{u}_{nn}}.$$
  It can be observed that in Eq. (59), we divide by a scalar value each time, eliminating the need for matrix inversion and greatly reducing the complexity. ${\delta }_{n}$ represents the *n*-th element of ${\varvec{\updelta}}$. ${\delta }_{n}$ can be expressed as:
  60 $${\delta }_{n}=\frac{1}{2{u}_{nn}}\left|2{V}_{n}-\sum_{l=1, l\ne n}^{N}{\overline{\theta }}_{l}({u}_{ln}+{u}_{nl})\right|,$$
  Based on Eq. (60), it can be seen that the obtained closed-form solution for RIS passive beamforming optimization no longer involves a matrix inversion process, and the division by ${u}_{nn}$ results in a scalar value. The remaining calculation steps involve scalar addition and subtraction. Computing the optimal closed-form solution for one element requires $N$ calculations, where RIS has $N$ elements, and the multiplication is performed $N\times N$ times, resulting in a computational complexity of $\mathcal{O}\left({N}^{2}\right)$.Therefore, based on the above derivation, the detailed steps of the low-complexity alternating optimization algorithm proposed are shown in Algorithm 1:

**Algorithm 1:**
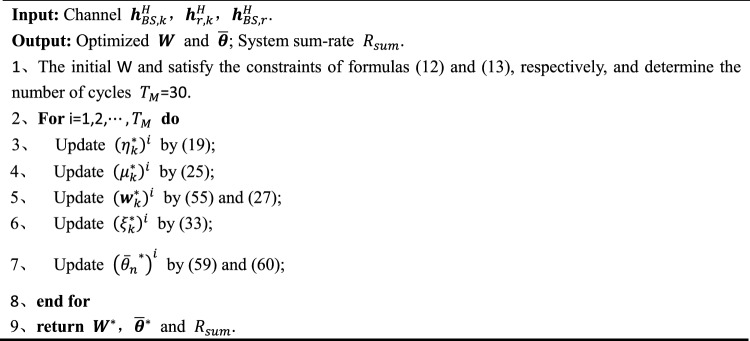
Proposed joint beamforming design.
3. Complexity analysis
  The computational complexity of calculating ${{\varvec{w}}}_{k}^{*}$ before transformation is $\mathcal{O}\left({M}^{3}\right)$, while the computational complexity of calculating ${{\varvec{w}}}_{k}^{*}$ after the "Woodbury transformation" is still $\mathcal{O}\left({M}^{2}\right)$. The computational complexity of calculating the optimized ${\overline{\theta }}_{n}$ using the ADMM algorithm is $\mathcal{O}\left({N}^{3}\right)$, and the computational complexity after scalar transformation is $\mathcal{O}\left({N}^{2}\right)$.Finally, the computational complexity of the traditional and proposed beamforming optimization algorithms is $\mathcal{O}\left({M}^{3}\right)+\mathcal{O}\left({N}^{3}\right)$ and $\mathcal{O}\left({M}^{2}\right)+\mathcal{O}\left({N}^{2}\right)$, respectively. In general, in communication systems, receivers are subject to noise, making it challenging to obtain accurate channel state information. Therefore, the estimated channel state information will inevitably have some errors. We can represent the simulated estimated channel ${\widetilde{{\varvec{H}}}}_{k}^{H}$ as:
  61 $${\widetilde{{\varvec{H}}}}_{k}^{H}={{\varvec{H}}}_{k}^{H}+e,$$
  where ${{\varvec{H}}}_{k}^{H}$ represents the true channel, and $e\sim \mathcal{C}\mathcal{N}\left(0,{\sigma }_{e}^{2}\right)$ represents the channel estimation error following a Gaussian distribution with zero mean. We assume that the variance of ${\sigma }_{e}^{2}$ satisfies ${\sigma }_{e}^{2}\triangleq \iota {\left|{{\varvec{H}}}_{k}^{H}\right|}^{2}$, where $\iota$ represents the ratio of channel estimation error power to channel gain ${\left|{{\varvec{H}}}_{k}^{H}\right|}^{2}$, reflecting the level of channel estimation error. We then apply the proposed algorithm to the ${\widetilde{{\varvec{H}}}}_{k}^{H}$ obtained through channel estimation errors. Due to limited space, we will not provide a detailed description of the algorithm here. Instead, we will replace ${{\varvec{H}}}_{k}^{H}$ in the derived algorithm from the previous section with ${\widetilde{{\varvec{H}}}}_{k}^{H}$. Therefore, the next section will present simulation results demonstrating the performance of the proposed algorithm.

## Results and discussion

**Figure 2:**
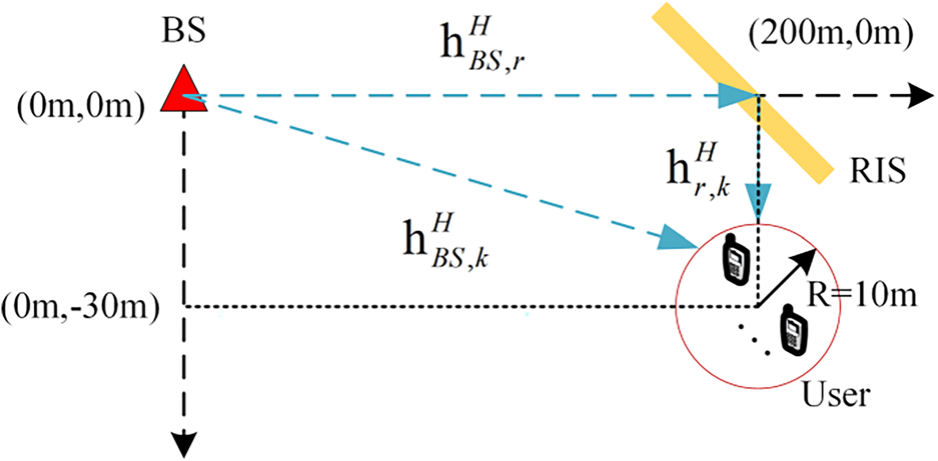
RIS assisted *K* user communication environment simulation.

In this section, we first present the simulation parameters. Then, based on the simulation results, we provide a detailed comparison of the performance between the proposed algorithm and existing algorithms under different conditions. As shown in Fig. 2, without loss of generality, we consider a base station with an average linear array (ULA) of *M* = 5 antennas and *N* = 80 reflective elements on the reconfigurable intelligent surface (RIS).The base station is located at coordinates (0 m, 0 m), and the RIS is located at (200 m, 0 m). There are *K* = 4 users randomly distributed within a radius of approximately 10 m around the coordinates (200 m, 30 m). The variance of the noise is defined as ${\upsigma }_{0}^{2}=1$, and the communication system has a bandwidth of 180 kHz. Additionally, the initially active beamforming vector ${\varvec{W}}$ satisfies $\text{trace}({\varvec{W}}{{\varvec{W}}}^{H})=P$, and the base station's transmit power $P$ is defined as ${10}^\frac{SNR}{10}$, where SNR represents the signal-to-noise ratio. The initial RIS coefficients satisfy the constraints in Eq. (13). We propose three schemes for comparison:

- Scheme 1: No optimization of RIS phase shifts, using random phase-shift reflection coefficients.
- Scheme 2: No assistance from RIS, only communication links between the base station and users.
- Scheme 3: ADMM algorithm with optimized phase shifts.

**Figure 3:**
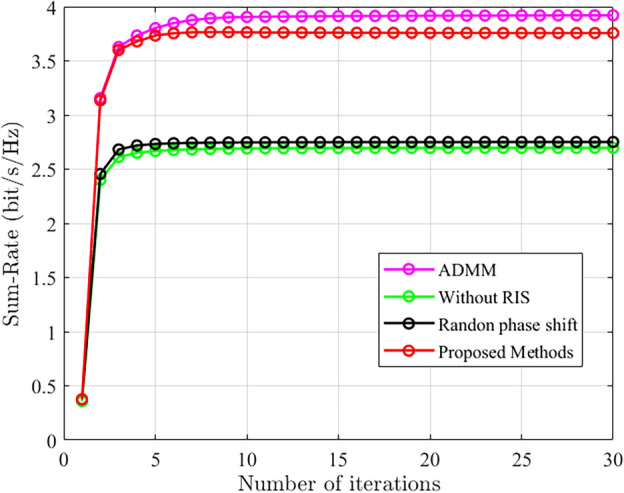
The convergence comparison between the proposed algorithm and other algorithms.

In this section, the performance of the proposed algorithm is comprehensively analyzed through simulation results. Figure 3 illustrates the convergence performance of the proposed algorithm compared to the other schemes. The simulation environment in Fig. 3 includes a base station with a transmit power of 1 dBm, 4 users, 5 base station antennas, and 80 elements deployed on the RIS. It can be observed from the figure that the proposed method has some loss in performance compared to Scheme 3, which may be due to suboptimal solutions during the "scalar" conversion process. However, the proposed algorithm greatly reduces the complexity of the optimization algorithm. Scheme 3 proposes a joint optimization of beamforming design and involves communication links between the base station and users. Scheme 1 optimizes the active beamforming design of the base station using the proposed method, and Scheme 2 optimizes the proposed method. Through analysis, it can be concluded that even without optimizing the phase shifts, the system can achieve good performance with RIS-assisted communication. It can also be observed that the proposed method achieves good convergence and relatively good performance under different communication conditions.

**Figure 4:**
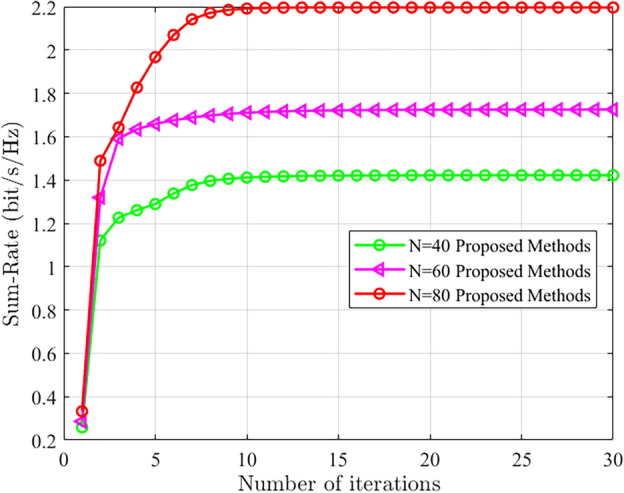
The convergence performance of the proposed scheme under different number of RIS elements is compared.

Figure 4 illustrates the comparison of system and rate performance of the proposed method under different numbers of RIS elements. The base station has a transmit power in $-5$ dBm, 5 base station antennas, 4 users, and the RIS has 80 reflect elements. It can be observed that as the number of RIS elements increases, both the system and rate performance improve. Additionally, it can be seen that the proposed method demonstrates good convergence performance.

**Figure 5:**
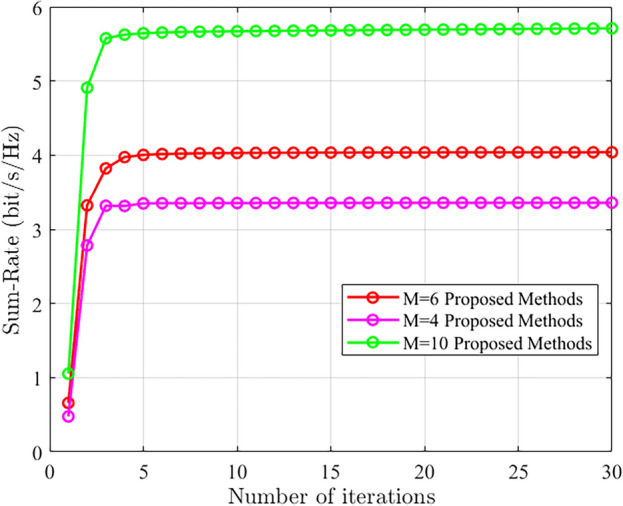
The convergence performance comparison of the proposed scheme under different number of base station antennas.

To further demonstrate the performance of the proposed method, convergence analysis was conducted under different numbers of base station antennas. Figure 5 shows the results when the transmit power is set to 0 dBm, the RIS has 80 elements, and there are 4 users. It can be observed that as the number of base station antennas increases, both the system performance and rate continuously improve. Moreover, the proposed algorithm exhibits good convergence performance at different numbers of base station antennas.

**Figure 6:**
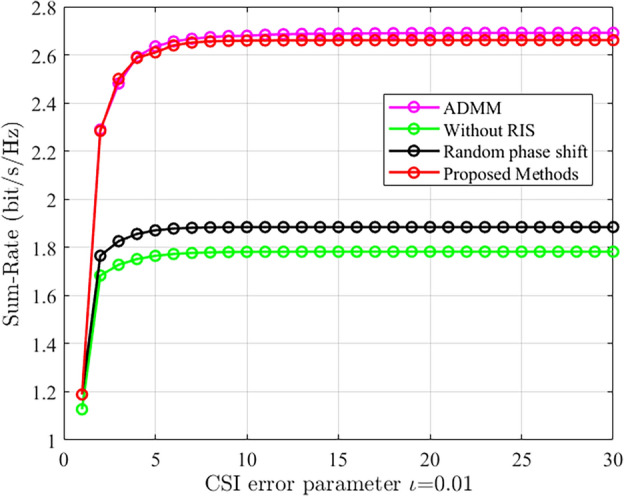
The convergence performance of the proposed scheme is compared with other schemes.

To validate the convergence performance of the proposed algorithm in the presence of CSI errors, convergence experiments were conducted. The simulation environment considered a transmit power of 0 dBm, 4 users, 5 base station antennas, 80 RIS elements, and a channel error parameter of 0.01. Figure 6 demonstrates that both the proposed algorithm and the baseline algorithms exhibit good convergence performance under the presence of CSI errors.

**Figure 7:**
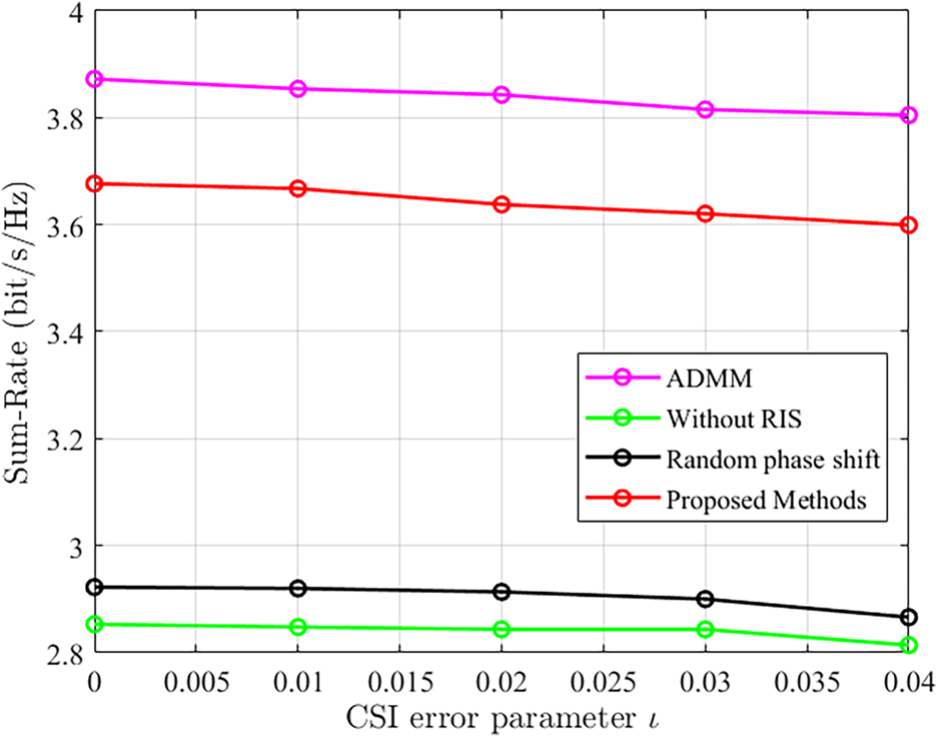
The comparison of system sum rate under different channel error parameters.

As shown in Fig. 7, as the channel parameter error increases, both system and rate exhibit a decreasing trend. However, since small error parameters were used in this experiment, the decreasing trend is not very significant. This also highlights the importance of obtaining accurate CSI in achieving high system performance and rate.

**Figure 8:**
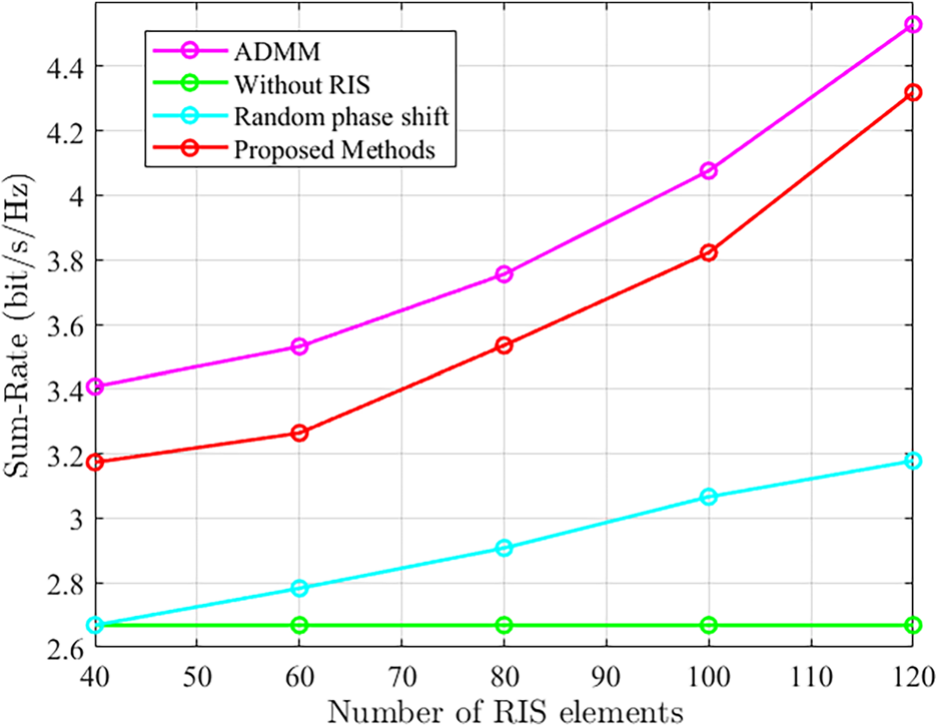
The comparison of system sum rate under different RIS elements.

As shown in Fig. 8, we further investigate the impact of increasing RIS elements on system and rate performance. We set the system parameters as $P=0$ dBm, 5 antennas, 4 users, and there is no channel error. It can be observed that as the number of RIS elements increases, both the system and rate continuously improve. Without RIS-assisted communication, the system and rate remain unchanged with the variation of RIS elements. The random phase shift optimization only focuses on optimizing the beamforming design at the active base station without optimizing the RIS phase shift design, which explains the relatively small variations in system and rate performance.

**Figure 9:**
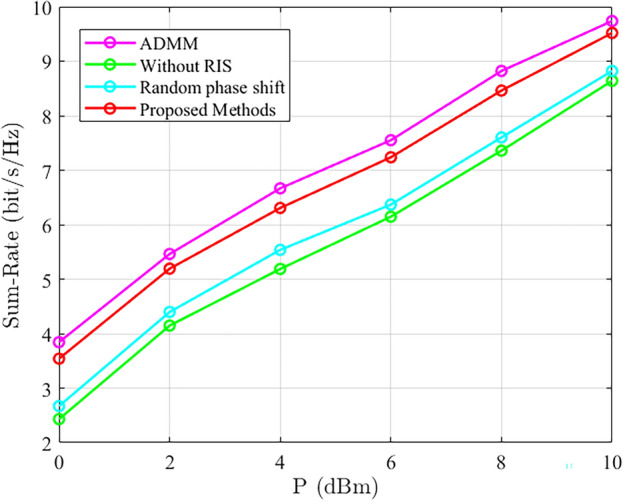
Comparison of system sum rate performance under different base station transmission power.

Figure 9 validates the system and rate performance of the proposed method and other methods under different base station transmit power conditions. We set the number of antennas at the base station to be 5 and the number of RIS elements to be 80. It can be observed that the proposed ADMM-based method achieves better system gain. In the case of random phase shift optimization, both system and rate are improved compared to systems without RIS-assisted communication.

## Conclusions

In this article, we considered a downlink communication system aided by RIS and formulated a non-convex optimization problem to maximize the system throughput. However, as the number of base station antennas and RIS elements grows, traditional algorithms become computationally complex. To address these issues, we first transformed the original problem using fractional programming into three sub-problems. Then, we introduced auxiliary variables in each sub-problem and derived low-complexity, closed-form solutions based on the Woodbury and scalar transformations. Simulation results show that the proposed method outperforms traditional algorithms.

## Data Availability

The data used during the current study available from the corresponding author on reasonable request.
